# Combined retinal proteome datasets in response to atropine treatment using iTRAQ and SWATH-MS based proteomics approaches in guinea pig myopia model

**DOI:** 10.1016/j.dib.2020.106526

**Published:** 2020-11-17

**Authors:** Ying Zhu, Jingfang Bian, Daqian Lu, Qiong Wang, Boteng Gong, King-Kit Li, Fengjuan Yu, Jimmy Ka-Wai Cheung, Xiaowen Ji, Hongmei Zhang, Bei Du, Hong Nian, Chi-ho To, Ruihua Wei, Thomas Chuen Lam

**Affiliations:** 1 Tianjin International Joint Research and Development Centre of Ophthalmology and Vision Science, Eye Institute and School of Optometry, Tianjin Medical University Eye Hospital, Tianjin, China; 2 Centre for Myopia Research, School of Optometry, The Hong Kong Polytechnic University, Hong Kong SAR, China

**Keywords:** Guinea pigs, FDM, Atropine, iTRAQ, SWATH

## Abstract

Atropine, a non-selective muscarinic antagonist, is known to slow down myopia progression in human adolescents and in several animal models. However, its underlying molecular mechanism is unclear. The present work built a monocular form-deprivation myopia (FDM) guinea pig model, using facemasks as well as atropine treatment on FDM eyes for 2 and 4 weeks. Retinal protein changes in response to the FDM and effects of topical administration of atropine were screened for the two periods using fractionated isobaric tags for a relative and absolute quantification (iTRAQ) approach coupled with nano-liquid chromatography-tandem mass spectrometry (nano-LC–MS/MS) (*n*=24, 48 eyes). Retinal tissues from another cohort receiving 4-weeks FDM with atropine treatment (*n*=12, 24 eyes) with more significant changes were subjected to sequential window acquisition of all theoretical mass spectra (SWATH-MS) proteomics for further protein target confirmation. A total of 1695 proteins (8875 peptides) and 5961 proteins (51871 peptides) were identified using iTRAQ and SWATH approaches, respectively. Using the Paragon algorithm in the ProteinPilot^TM^ software, the three most significantly up-regulated and down-regulated proteins that were commonly found in both ITRAQ and SWATH experiments are presented. All raw data generated from the work were submitted and published in the Peptide Atlas public repository (http://www.peptideatlas.org/) for general release (Data ID PASS01507).

## Specifications Table

**Table:** 

Subject	Ophthalmology
Specific subject area	Retinal proteome in guinea pig after atropine treatment
Type of data	Table, Graph, Figure
How data were acquired	iTRAQ labeled and SWATH-MS using Quadrupole Time-of-Flight TripleTOF 6600 mass spectrometer (SCIEX); Searched against the UniProt database (organism ID: 10141)
Data format	Raw, Analyzed
Parameters for data collection	Three week old guinea pigs were divided into four groups with body weight, refractive error, and axial length measured at the baseline and at several experimental time points up to 7 weeks. They were normal control group (NC), with monocular form deprivation myopia group (FDM), monocular FDM with atropine eye gel treatment for 2 weeks group (A1), and monocular FDM with atropine eye gel treatment for 4 weeks (A2). Animals were sacrificed at 7 weeks with retina tissues collected for both labeled and label-free proteomics analysis.
Description of data collection	Retinas of guinea pigs were pooled as representing groups for NC, FDM, A1, and A2 groups, followed by isobaric tags for a relative and absolute quantification (iTRAQ) 8-plex labeling with an equal amount of proteins from the four groups. After mixing, the combined lysate mixture of each group was divided into five SCX fractions, followed by nano LC-MS/MS, using an Eksigent ekspert™ nanoLC 425 system coupled to TripleTOF 6600 System (SCIEX). Another cohort of FDM and A2 groups were injected individually for confirmation of differentially expressed proteins in response to 4-week atropine treatment. A retinal spectral library was generated from the pooled samples using information dependent acquisition (IDA) and relative quantification was performed using an independent data acquisition (SWATH-MS) approach.
Data source location	Centre for Myopia Research, School of Optometry, the Hong Kong Polytechnic University, Kowloon, Hong Kong Tianjin International Joint Research and Development Centre of Ophthalmology and Vision Science, Eye Institute and School of Optometry, Tianjin Medical University Eye Hospital, Tianjin, China
Data accessibility	Repository name: Peptide Atlas Data identification number: [PASS01507] Official URL for this dataset: http://www.peptideatlas.org/PASS/PASS01507 Direct URL to data: [ftp://PASS01507:y1017so@ftp.peptideatlas.org/] To access files via FTP, use credentials: Servername: ftp.peptideatlas.org Username: PASS01507 Password: y1017so

## Value of the Data

• The dataset describing a comprehensive retinal proteome identified in the guinea pig FDM model, using a high-resolution proteomics approach.
• This work explored the feasibility of using combined iTRAQ LC-MS/MS and SWATH-MS protocols in quantifying protein changes and potential mechanism(s) of atropine in myopia control treatment for eye research.
• The publicly available retinal spectral library generated provides an important new resource for future myopia studies or eye research using a data-independent acquisition (DIA) approach. Differentially expressed protein identified in the study provides new evidence of key molecular events in myopic guinea pigs in response to atropine treatment.

## 1 Data Description

In the current work, combined proteomics approaches, iTRAQ LC-MS/MS, and SWATH-MS were used to screen and verify differential retinal proteins in response to the atropine treatment in the guinea pig FDM [1], which is more relevant to human myopia.

**Fig. 1:**
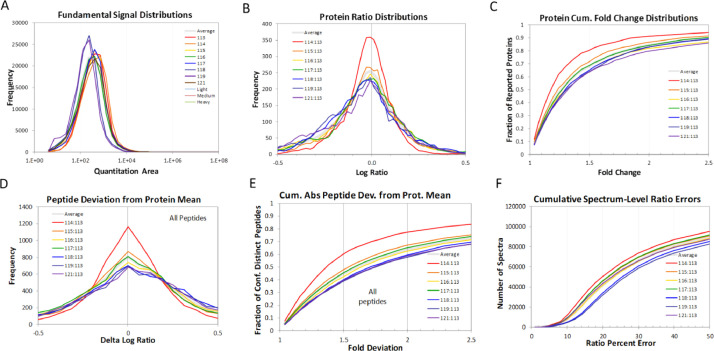
Quantification metrics of different isobaric tags in iTRAQ data. (A) Fundamental signal distribution. (B) Distribution of protein ratio. (C) Distribution of protein cumulative fold change. (D) Peptide deviation from protein mean. (E) Cumulative peptide deviation from protein mean. (F) Cumulative spectrum-level ratio errors.

**Fig. 2:**
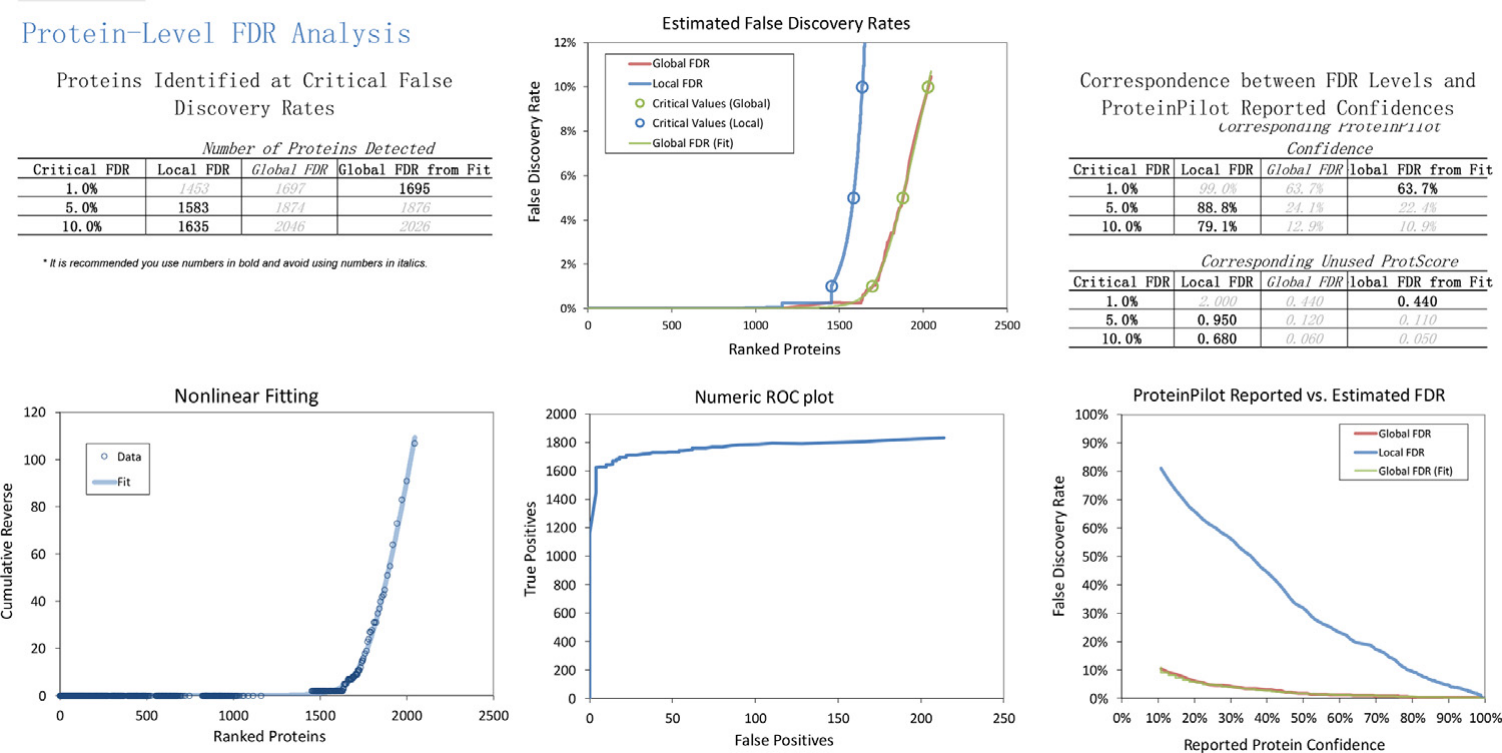
Protein level FDR analysis of guinea pig retinal proteome identified by ProteinPilot^TM^ software using iTRAQ approach

**Fig. 3:**
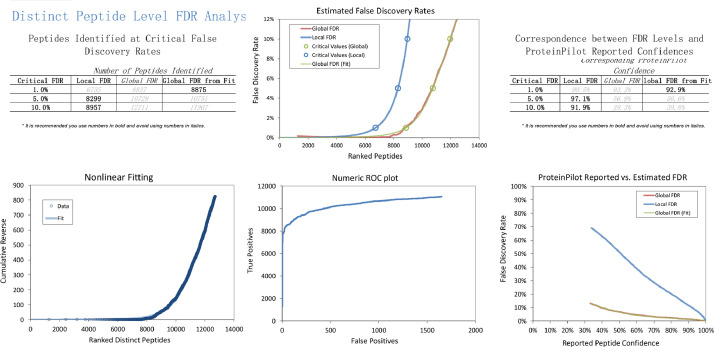
Peptide level FDR analysis of guinea pig retinal proteome identified by ProteinPilot^TM^ software using iTRAQ approach.

**Fig. 4:**
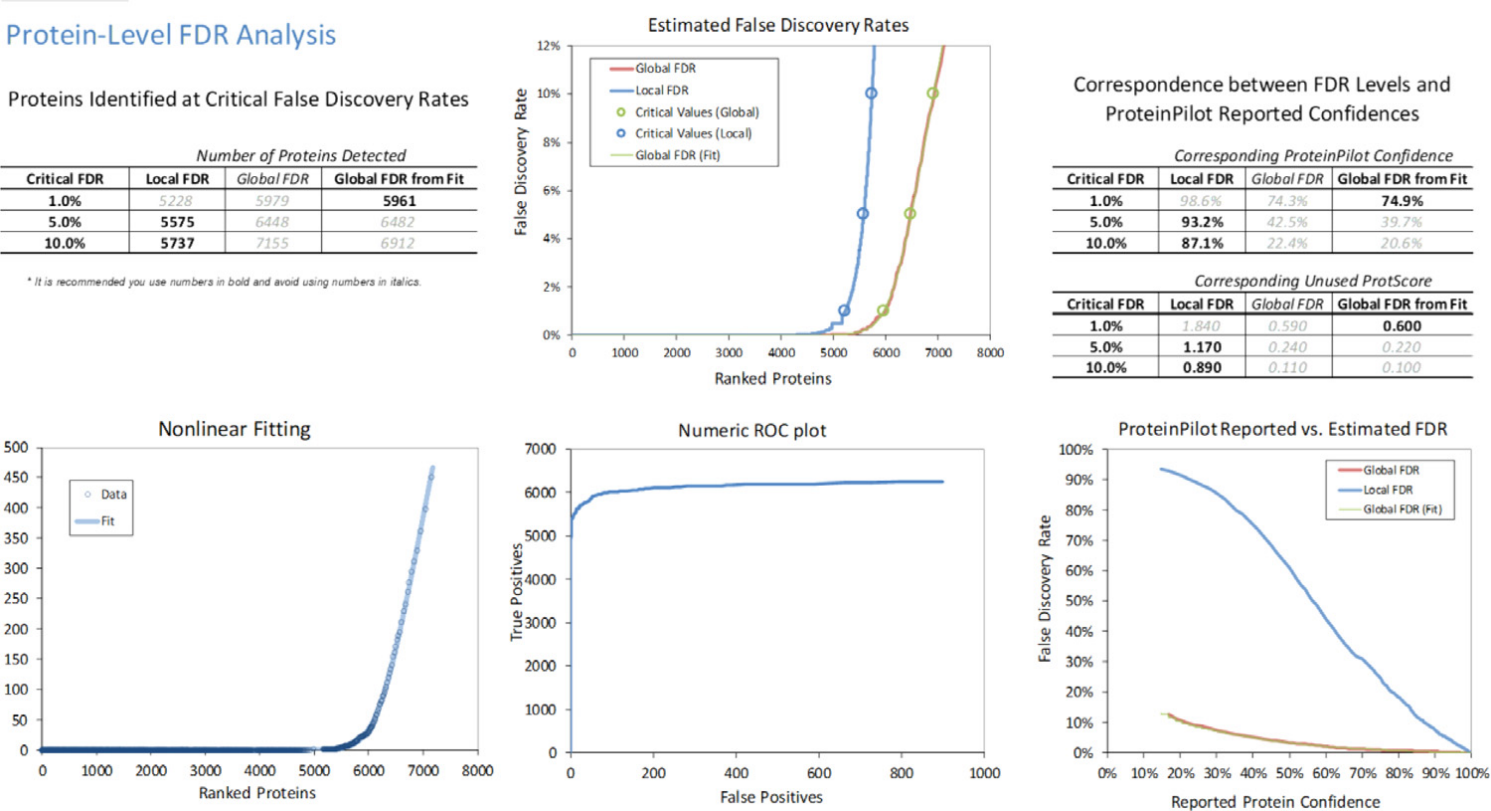
Protein level FDR analysis of retinal IDA library generated by ProteinPilot^TM^ software for SWATH-MS.

**Fig. 5:**
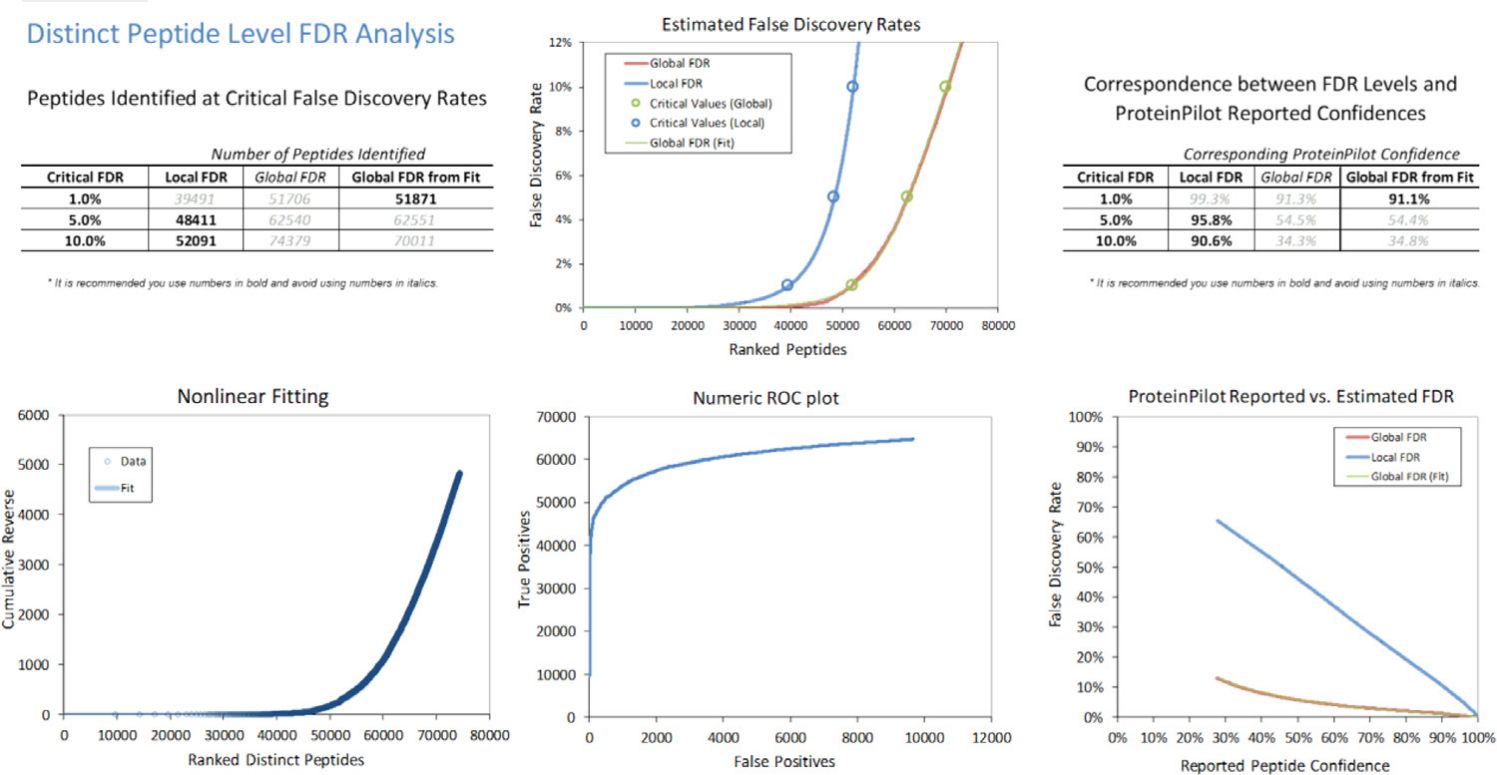
Peptide level FDR analysis of retinal IDA library generated by ProteinPilot^TM^ software for SWATH-MS.

In the iTRAQ LC-MS/MS approach, retinal lysates of age-matched animals from each of the normal control (NC), monocular form deprivation myopia (FDM), FDM with 2 weeks atropine treatment (A1) and FDM with 4 weeks treatment (A2) groups were pooled together for MS analysis. To evaluate the quality of iTRAQ data, the signal distribution of each labeled group, distribution of protein ratio, peptide deviation from protein mean, and cumulative spectrum level ratio errors were determined and shown in Fig. 1**.** In addition, the characteristics of the trypsin digestion spectrum are shown in **Fig. S1** with the associated raw data shown in **Table. S3.** The overall mass distribution, precursor charge and mass accuracy histograms are shown in **Fig. S2**. For the SWATH-MS approach, age-matched individual retinal samples of FDM and FDM with 4 weeks atropine treatment groups (A2) were used. All retinal samples from FDM and A2 groups were pooled respectively to form one representative lysate followed by high pH reversed-phase fractionation into fractions to generate a master spectral library by Information Dependent Acquisition (IDA). Protein identification was performed with ProteinPilot ^TM^ (v5.0, SCIEX). For data independent acquisition (SWATH-MS), individual biological samples (2 μg each) with two technical replicates were analyzed separately. Fig. 2 and Fig. 3 show the FDR analysis results of proteins and peptides respectively found by iTRAQ LC-MS/MS. A total of 1695 non-redundant proteins (8875 peptides) were identified **(Table. S1),** with 1% global FDR values. Fig. 4 and Fig. 5 show the proteins and peptides analysis results of SWATH-MS. A total of 5961 non-redundant proteins (51871 peptides) were identified in a combined library **(Table. S2)** with 1% global FDR. Table. 1 shows three typical up-regulated and down-regulated differentially identified proteins found by iTRAQ LCMS and SWATH-MS (≥ 1 peptide per protein, fold change ≥ 1.50 in both iTRAQ LC-MS-MS and SWATH-MS with *P* < 0.05).

**Table 1:** Examples of three up-regulated and three down-regulated proteins found by both iTRAQ LC-MS/MS and SWATH-MS in response to 4-wk (A2) atropine *vs.* FDM with expression ratio, P-value.

Uniprot ID	Gene name	Protein name	iTRAQ ratio (A2/FDM)	SWATH ratio (A2/FDM)	SWATH p-value
**Up-regulated proteins:**					
H0VFF0	Rps7	40S ribosomal protein S7	5.15	1.67	0.00
A0A286Y4G4	KRT3	IF rod domain-containing protein	4.97	1.95	0.03
H0V362	SLC4A7	Anion exchange protein	3.40	1.57	0.01
**Down-regulated proteins:**					
H0UZK2	SNAP25	Synaptosomal-associated protein	0.67	0.23	0.00
H0W051	SNCG	Gamma-synuclein	0.56	0.49	0.00
A0A286XMP4	HNRNPD	Heterogeneous nuclear ribonucleoprotein D	0.55	0.51	0.01

## 2 Experimental design, materials, and methods

### 2.1 Animals

Twenty-nine pigmented male Guinea pigs (Cavia procellus, the English short-hair stock) were raised with their mother till postnatal day 18 for experiment starting from postnatal 3 weeks to 7 weeks. The FDM model was established based on a published protocol [2]. Guinea pigs were raised in standard cages (65 × 45 × 20 cm) at 25 °C with sufficient food, water, and fresh vegetables daily. The luminance in the center of each cage was approximately 300 lux, provided by straight fluorescent lamps under a daily 12-hour light / 12 h dark cycle (starting at 8:00 AM). Guinea pigs were randomly assigned to four groups: normal control group (NC, *n*=7), monocular form deprivation myopia group (FDM, *n*=7), FDM with 2-week atropine-treated group (A1, *n*=7) and FDM with 4-week atropine-treated group (A2, *n*=8). For the NC animals, both of eyes were exposed naturally without any treatment, for the FDM group, either the right or left eye was covered by a white latex facemask, leaving the contralateral eye, nose, mouth, and ears freely exposed from 3 weeks to 7 weeks old. In the atropine treatment groups (A1 and A2), atropine gel (10 g• L-1) was topically administered to the FDM eyes with for 2- or 4-weeks, respectively. Refractive error and axial length were measured using a streak retinoscope (66Vision, China) and A-Scan (Kangning, China). Respectively. across the experimental period. For the validation test, a further cohort of guinea pigs (*n*=12) with FDM and atropine treatment for 4 weeks (A2) were collected.

### 2.2 Retina collection and protein extraction

The guinea pigs were sacrificed by cervical vertebra dislocation after ocular biometrics measurement at 7 weeks. After removing the connective tissue attached to eyeball, anterior segment, crystalline lens, and vitreous from the eye cup, the retina was peeled off carefully from the posterior hemisphere without retinal pigment epithelium and frozen immediately. After homogenization and protein assay measurement, six individual samples with sufficient and similar protein concentrations from each group were selected for subsequent proteomics analysis.

### 2.3 iTRAQ labelling

For iTRAQ LC-MS/MS, 200 µl of Nitroextra (9M Urea, SDS, Tritone X, and Protease inhibitors) was added into each frozen tissue. Samples were pooled and blended using a homogenizer and sonicated for 5 min. Protein in the solution was purified by adding it to three sample volumes of pre-cooled acetone (320110, Sigma) and incubating at -20 °C overnight. Precipitated proteins were then collected by centrifuging at max speed (∼14 k RPM) for 15 min. The protein pellet was washed twice with an equal volume of pre-cooled acetone. Residual acetone was removed by drying in a biosafety cabinet. Proteins were resuspended in 8 M urea and reduced with 20 mM dithiothreitol (DTT) at 60 °C for 1 h, then alkylated with 40mM iodoacetamide at room temperature for 30 min protected from light. 10 mM DTT was used to quench the alkylation reaction. Samples were diluted to 2 M urea with HPLC grade water and digested with trypsin in 1:100 (w: w) ratio at 37 °C overnight.

A 100 µg aliquots of pooled desalted peptides from each experimental condition were chemically labeled with iTRAQ 8-plex reagent (4466096, SCIEX) in 100 mM TEAB As follows: 113: right eyes of NC; 114: left eyes of NC; 115: fellow control eyes of A1; 116: treatment eyes of A1; 117: fellow control eyes of A2; 118: treatment eyes of A2, 119: fellow control eyes of FDM; 121: treatment eyes of FDM. In short, an appropriate amount of isopropanol was added to each iTRAQ reagent tube, such that when combined with each sample there was a minimum concentration of 70% isopropanol. Each reagent tube was well mixed by vortexing. The pH of the combined sample was checked again to ensure the final pH was between pH7-10, or if not, adjusted with 1 M.

Triethylammonium bicarbonate (TEAB) is added to adjust the pH. The labeling reaction was allowed to perform at room temperature for 2 h. All the labeled samples were then dried for C18 desalting. Differentially labeled peptide samples were re-suspended in SCX buffer A (10mM KH2PO3, 20% acetonitrile (ACN), pH2.7). SCX chromatography was performed with a PolySULFOETHY ATM (200 × 4.6 mm, 200 A) column using step gradients (0–10 min: 0%B, 29 min: 15%B, 44 min: 45%B, 46–53 min: 100%B) of Buffer A and B (10mM KH2PO3, 20%ACN, 0.6M KCl, pH2.7) and a flow rate of 1 min/ml. In total 53 × 1 ml fractions were collected. They were further combined into five fractions based on the number of protein identified in each 10 min fraction. Each fraction was desalted with ZipTip (Cat. ZTC18S960, Millipore) and dried in a spin vacuum for LC-MS/MS analysis.

### 2.4 LC-MS/MS Analysis for iTRAQ

Each dried peptide sample was dissolved in 12 ul of 0.1% FA. About 3 µg fractionated samples were separated by LC-MS/MS using an Eksigent ekspert™ nanoLC 425 system coupled to a TripleTOF 6600 System (SCIEX). After the sample was loaded, peptides were trapped (ChromXP nanoLC Trap column 350 μm × 0.5 mm, ChromXP C18 3 μm) and eluted at a flow rate of 300 nL/min into a reverse phase C18 column using a linear gradient of ACN (3–36%) in 0.1% formic acid with a total run time of 120 min, including mobile phase equilibration. Mass spectra and tandem mass spectra were recorded in positive-ion and “high-sensitivity” mode with a resolution of ∼35,000 full-width half-maximum. The nanospray needle voltage was typically 2,300 V in HPLC-MS mode. After the acquisition of about 5 to 6 samples, TOF MS spectra and TOF MS/MS spectra were automatically calibrated during dynamic LC-MS & MS/MS auto-calibration acquisitions injecting 25 fmol alcohol dehydrogenase. For collision induced dissociation tandem mass spectrometry (CID MS/MS), the mass window for precursor ion selection of the quadrupole mass analyzer was set to ± 2 m/z. The precursor ions were fragmented in a collision cell using nitrogen as the collision gas. Advanced IDA was used for MS/MS collection on the Triple TOF 6600 to obtain MS/MS spectra for the 20 most abundant and multiple charged (*z* = 2, 3 or 4) following each survey MS1 scan allowing typically for 250 ms acquisition time per each MS/MS. Dynamic exclusion is set for 30 s after 2 repetitive occurrences.

ProteinPilot^TM^ (v5.0, SCIEX) was used for iTRAQ analysis. The error tolerance for precursor mass was 15.0 ppm and fragment ion 0.2 Da. Decoy database was used to control the false discovery rate (FDR) at 1%. Filters of parameters were set as follows: fold change (≥ 1.50), and at least 1 matched peptide achieving 95% confidence per protein.

### 2.5 SWATH-MS analysis

For SWATH-MS, 200μl of SDS lysis buffer (5% SDS, 50mM TEAB, Ph7.55) was added into each frozen tissue. Samples were homogenized at 5800rpm, 4 °C for 4 × 30 s cycles, with an interval of 20 s between cycles (Precellys Evolution, Bertin Instrument). They were then centrifuged at 15,000rpm, 4 °C for 30 min and the supernatants collected. Proteins were reduced with 20 mM DTT at 95 ^°^C for 10 min, then alkylated with 40 mM IAA at room temperature for 10 min with protection from light, before addition of 1.2% phosphoric acid to quench the alkylation reaction. Samples were added to S-Trap protein binding buffer and proteins were trapped by a filter in the S-Trap Micro Spin Column (Protifi, U.S.) [3]. After trypsin (Promega, U.S.) digestion in 1:25 (w/w, trypsin: protein) ratio at 47 ^ο^C for 1 h, peptides were eluted with 50 mM TEAB and 0.2% aqueous formic acid (FA), then peptides were recovered with elution of 50% ACN containing 0.2% FA and dried by vacuum centrifuge at 4 °C. The peptides were resuspended with 0.1% FA for LC-MS/MS analysis (calibrated at 0.5 μg/μl) using Pierce Quantitative Colorimetric Peptide Assay (Thermo Fisher Scientific, U.S.).

Both information-dependent acquisition (IDA) and SWATH-MS analyses were performed by the TripleTOF 6600 system (SCIEX) connected to an Eksigent ekspert™ nanoLC415 system similar to our previous protocols [4], [5] In both IDA and SWATH acquisitions, a digested sample (2 µg) was loaded to a trap column (100 µm x 2 cm, C18) at 2 µl/ min for 15 min by loading buffer (0.1% FA, 2 % ACN in water). It was then separated on a nano-LC column (100 µm x 30 cm, C18, 5 µm). LC separation was performed under 350 nL/min using mobile phase A (0.1% FA + 2 % ACN in water) and B (0.1% FA, 98% ACN in water) with the following gradient: 0–0.5 min: 5%B, 0.5–90 min:10%B, 90–120 min:20%B, 120–130 min:28%B, 120–135 min:45%B, 135–141 min:80%B, 141–155 min:5%B. An isolation of 100 Variable windows were selected in a looped mode over the full mass range of 100- 1800 m/z scan in SWATH acquisition.

### 2.6 Ion library generation for SWATH analysis

The retina tissues of the 12 guinea pigs (FDM and A2 groups, *n*=6 per group) were pooled together and then divided into 6 fractions using High pH Reversed-Phase peptide fractionation kit. Six separate IDA injections were combined to generate an ion library (.group file) for SWATH analysis. It was searched against the guinea pig Uniprot database in ProteinPilot^TM^ (v5.0, SCIEX) software utilizing the Paragon algorithms with the following parameters: identification Sample Type, iodoacetamide Cys Alkylation, trypsin digestion, thorough search effort and with FDR analysis. The resulting ProteinPilot^TM^ group file was used as the ion library file for all SWATH file processing and quantification.

### 2.7 SWATH-MS acquisitions and processing

Two micrograms (2 µg) of all 24 biological samples (both eyes of FDM group and A2 group) with two technical replicates each were injected for SWATH-MS quantification. The generated raw data (.wiff) were processed with PeakView (V2.2, SCIEX) to extract relevant transitions of each iden+tified peptide/protein using the generated combined ion library. Fifteen peptides with high signal/noise ratios were selected for retention time calibration. Resulting data were exported to MarkerView (V1.3.1, SCIEX) for normalization using the MLR method [6], followed by statistical analysis. Proteins which met the criteria: fold change ≥ 1.50, *P*-value of ≤ 0.05 (welch T-test) were considered to be significant changes.

## Ethics Statement

All procedures were carried out in accordance with the regulations stipulated by the Animal Use and Protection Committee at Tianjin Medical University and conformed to The Association for Research in Vision and Ophthalmology Statement for the Use of Animals in Ophthalmic and Vision research.

## Declaration of Competing Interest

The authors declare that they have no known competing financial interests or personal relationships which have, or could be perceived to have, influenced the work reported in this article.

## Appendix Supplementary materials

Image, image 1

Image, image 2

Image, application 3

Image, application 4

Image, application 5
