# Supplementary figures and images for: Combined retinal proteome datasets in response to atropine treatment using iTRAQ and SWATH-MS based proteomics approaches in guinea pig myopia model
Source: Data Brief. 2020 Nov 17;33:106526. doi: 10.1016/j.dib.2020.106526 (PMC7708793; doi:10.1016/j.dib.2020.106526)

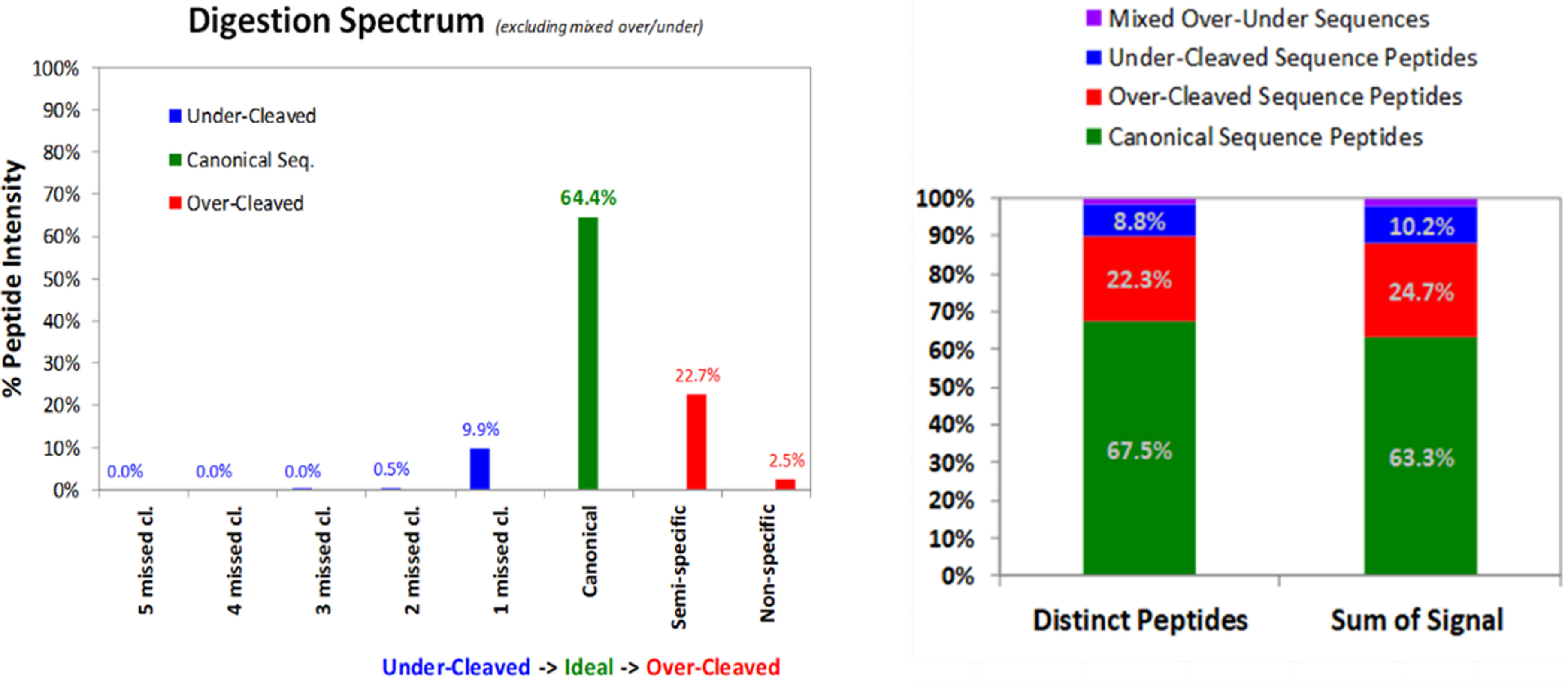

Supplement: Supplementary file 1 [file mmc1.jpg]

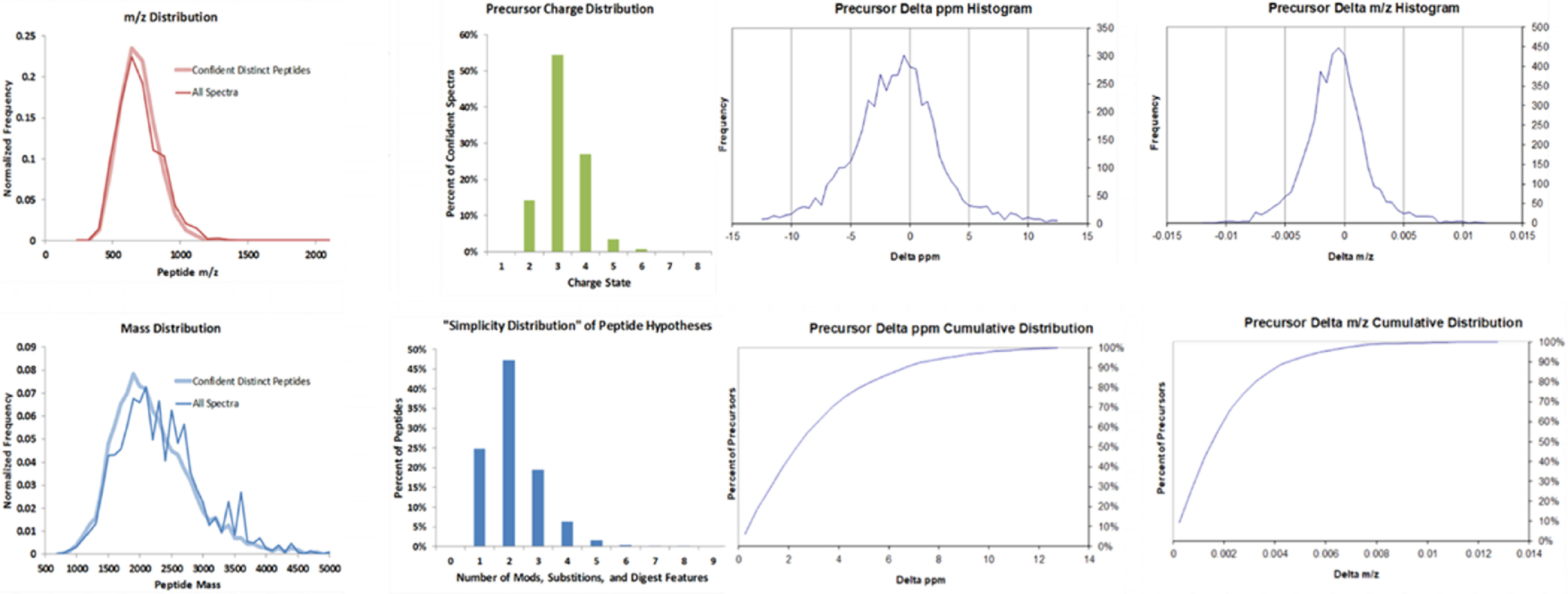

Supplement: Supplementary file 2 [file mmc2.jpg]
